# Extracellular Vesicles From the Helminth *Fasciola hepatica* Prevent DSS-Induced Acute Ulcerative Colitis in a T-Lymphocyte Independent Mode

**DOI:** 10.3389/fmicb.2018.01036

**Published:** 2018-05-23

**Authors:** Javier Roig, Maria L. Saiz, Alicia Galiano, Maria Trelis, Fernando Cantalapiedra, Carlos Monteagudo, Elisa Giner, Rosa M. Giner, M. C. Recio, Dolores Bernal, Francisco Sánchez-Madrid, Antonio Marcilla

**Affiliations:** ^1^Àrea de Parasitologia, Departament de Farmàcia i Tecnologia Farmacèutica i Parasitologia, Universitat de València, Burjassot, Spain; ^2^Facultad de Ciencias de la Salud, Universidad Europea de Valencia, Burjassot, Spain; ^3^Vascular Pathophysiology Area, Centro Nacional de Investigaciones Cardiovasculares, Madrid, Spain; ^4^Joint Research Unit on Endocrinology, Nutrition and Clinical Dietetics, Health Research Institute La Fe, Universitat de València, Burjassot, Spain; ^5^Veterinari de Salut Pública, Centre de Salut Pública de Manises, Burjassot, Spain; ^6^Departament de Patologia, Universitat de València, Burjassot, Spain; ^7^Departament de Farmacologia, Universitat de València, Burjassot, Spain; ^8^Departament de Bioquímica i Biologia Molecular, Universitat de València, Burjassot, Spain; ^9^Immunology Service, Hospital de La Princesa, Instituto de Investigación Sanitaria Hospital Universitario de La Princesa, Universidad Autónoma de Madrid, Madrid, Spain; ^10^Centro de Investigación Biomédica en Red Enfermedades Cardiovasculares, Madrid, Spain

**Keywords:** inflammatory bowel disease, DSS-ulcerative colitis, *Fasciola hepatica*, extracellular vesicles

## Abstract

The complexity of the pathogenesis of inflammatory bowel disease (ulcerative colitis and Crohn’s disease) has led to the quest of empirically drug therapies, combining immunosuppressant agents, biological therapy and modulators of the microbiota. Helminth parasites have been proposed as an alternative treatment of these diseases based on the hygiene hypothesis, but ethical and medical problems arise. Recent reports have proved the utility of parasite materials, mainly excretory/secretory products as therapeutic agents. The identification of extracellular vesicles on those secreted products opens a new field of investigation, since they exert potent immunomodulating effects. To assess the effect of extracellular vesicles produced by helminth parasites to treat ulcerative colitis, we have analyzed whether extracellular vesicles produced by the parasitic helminth *Fasciola hepatica* can prevent colitis induced by chemical agents in a mouse model. Adult parasites were cultured *in vitro* and secreted extracellular vesicles were purified and used for immunizing both wild type C57BL/6 and RAG1^-/-^ mice. Control and immunized mice groups were treated with dextran sulfate sodium 7 days after last immunization to promote experimental colitis. The severity of colitis was assessed by disease activity index and histopathological scores. Mucosal cytokine expression was evaluated by ELISA. The activation of NF-kB, COX-2, and MAPK were evaluated by immunoblotting. Administration of extracellular vesicles from *F. hepatica* ameliorates the pathological symptoms reducing the amount of pro-inflammatory cytokines and interfering with both MAPK and NF-kB pathways. Interestingly, the observed effects do not seem to be mediated by T-cells. Our results indicate that extracellular vesicles from parasitic helminths can modulate immune responses in dextran sulfate sodium (DSS)-induced colitis, exerting a protective effect that should be mediated by other cells distinct from B- and T-lymphocytes.

## Introduction

Ulcerative colitis and Crohn’s disease are two major clinical entities included in IBD, which may affect 0.5% of Western world population (1 million people in the United States, and 2.5–3 million people in Europe), with 37 new cases per 10^5^ inhabitants in Europe (Molodecky et al., 2012; Kaplan and Ng, 2017). IBD is also spreading in developing countries, where the prevalence is lower than in developed ones (Molodecky et al., 2012; Ng et al., 2013). Most of IBD patients are affected in their productive age, originating not only important social problems, but also economic losses (Kaplan, 2015).

The etiology of the disease is not well understood and because of the complexity of this pathology, the development of effective treatments will require studies using multidisciplinary approaches, where animal models have proved to be highly informative (Lin and Hackam, 2011).

Due to the absence of a curative treatment for IBD, there have been numerous attempts to incorporate new therapies. Some of these include new drugs or nutritional supplements to correct or enhance the effectiveness of the currently authorized treatments. In fact, the complexity of the pathogenesis of these diseases has led to empirically evolved current drug therapies, which primarily treat inflammation. The latest therapies attempt to restore the intestinal immune balance by efficiently combining immunosuppressant agents, biological therapy and modulators of the microbiota (Vanhove et al., 2016).

Interestingly, previous epidemiologic studies and clinic trials suggested that helminths, either by natural or artificial infections, might protect people from IBD (Smallwood et al., 2017). This has been confirmed by Ramanan et al. (2016), which have demonstrated that alteration of commensal and pathogenic bacteria produced by gastrointestinal helminths infection in turn protects the host against IBD (Ramanan et al., 2016). Still, major concerns of the helminthic therapy are related to ethical issues and the ability of controlling the course of the infection. In this sense, the identification of helminth-derived molecules that ultimately mediate host immune modulation is attracting much attention.

Extracellular vesicles, including exosomes and microvesicles, have been described as participating in intercellular communications with important roles in physiological and pathological processes, where they can be used as diagnostic and therapeutic weapons (Yáñez-Mó et al., 2015; Barile and Vassalli, 2017). EVs have been described to participate in inflammation, like EVs from granulocytes, which have been used for IBD treatment in a mice model (Wang et al., 2016; Song et al., 2017).

*Fasciola hepatica* is a flatworm that excretes/secretes a large number of molecules to the host-parasite interplay, including immune modulators (Dalton et al., 2013). Our group has identified some of these molecules in EVs released by parasite adults in culture (Marcilla et al., 2012; Cwiklinski et al., 2015; Fromm et al., 2015). To assess the functional role of these EVs in the host and exploring their usefulness as therapeutic agents against IBD, we have studied their potential in preventing ulcerative colitis in a DSS induced colitis model in C57BL/6 mice. Our data support that *F. hepatica* EVs (FhEVs) can protect from IBD in this model, since their inoculation prevents intestinal damage in acute colitis by altering the local immune response. Thus, FhEVs might be employed for preventing relapses in IBD patients and could be explored as a potential new therapy for treating IBD.

## Materials and Methods

### Materials

Unless otherwise specified, all reagents were purchased from Sigma-Aldrich (Madrid, Spain) and Bio-Rad Laboratories (Madrid, Spain). DSS, colitis grade (36–50 kDa) was purchased from MP Biomedical (United States). Specific antibodies for COX-2, p38 and p65 subunit nuclear factor-κβ (NF-κB) were purchased from Millipore (Billerica, MA, United States); antibodies against p38 MAPK and P-p38 MAPK were obtained from were obtained from Cell Signaling Technology (Danvers, MA, United States) and Santa Cruz Biotechnology (Santa Cruz, CA, United States), respectively. Anti-GAPDH polyclonal sera was kindly provided by Dr. Daniel Gozalbo, Universitat de València. ELISA kits for cytokines were purchased from Affymetrix eBioscience (San Diego, CA, United States).

### Isolation and Characterization of *Fasciola hepatica* Extracellular Vesicles

Extracellular vesicles from *F. hepatica* adults (FhEVs) were purified and monitored by TEM as previously described (Marcilla et al., 2012). Briefly, adult parasites, collected from cow livers from local abattoirs, were thoroughly washed with PBS and cultured in 0.2 μm filtered RPMI -1640 culture medium containing 100 U penicillin and 100 μg/mL streptomycin (all from Sigma), at concentrations of 2 worms/mL at 37°C for 5 h. After the incubation period, the parasite culture media was collected and centrifuged at low speed (first at 300 g/10 min, and then at 700 g/30 min) to remove larger debris, and the resulting supernatant was centrifuged at 15,000 *g* for 45 min at 4°C. Supernatants were then filtered using an ultrafiltration membrane (0.2 μm; Schleicher and Schuell) and centrifuged at 120000 *g*/1 h at 4°C in an Optima TL100 tabletop ultracentrifuge (Beckman) using a TLA-55 rotor.

Extracellular vesicles were aliquoted, frozen-drying in PBS containing 20% sucrose, and kept at 4°C until use. Before inoculating animals, protein content was determined following Bradford’s method (Bio-Rad).

### Animals

Female C57BL/6 mice (Harlan Interfauna Iberica, Barcelona, Spain), 6–8 weeks of age, weighing 18–20 g were previously acclimatized in 12 h light/dark cycles at 22°C and 60% humidity, for 7 days before performing the experiments, and fed with a standard laboratory rodent diet and water *ad libitum.* All animal care and experimental protocols were approved by the Institutional Ethics Committee of the Universitat de València and Generalitat Valènciana, Spain (No. 2015/VSC/PEA/00045 type 2, 12-03-2015).

Rag1^-/-^ mice were housed in pathogen-free conditions at the CNIC animal facility. Experimental procedures were approved by the local research ethics committee and conformed to EU Directive 2010/63EU and Recommendation 2007/526/EC, enforced in Spanish law under Real Decreto 53/2013.

### Induction of Dextran Sulfate Sodium (DSS) Colitis and Inoculation With FhEVs

**FIGURE 1:**
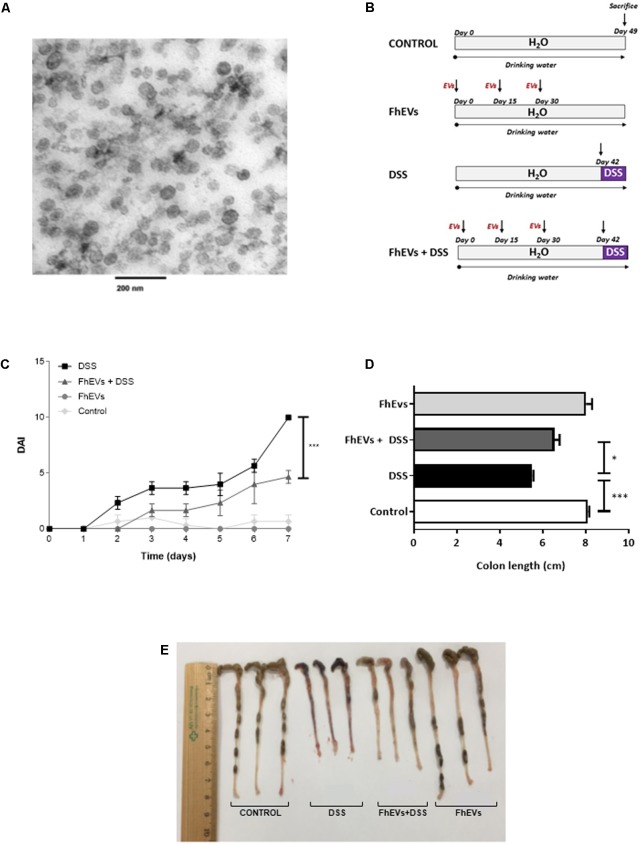
Treatment with *Fasciola hepatica* EVs ameliorates clinical symptoms and partially avoids colon shortening in DSS-induced acute colitis. **(A)** Extracellular vesicles were obtained by differential ultracentrifugation and ultrastructure was confirmed by TEM. **(B)** Schematic time schedule of immunization with *F. hepatica* EVs (FhEVs) and DSS-induction of colitis in C57B/L6 mice. **(C)** Disease activity index (DAI) was evaluated daily using the parameters of weight loss, diarrhea, and bleeding as described in methods. Statistical significance between two experimental groups was assessed using the Independent-Sample *t*-test (^∗∗∗^*p* < 0.001, significantly different between the control and DSS group; ^∗^*p* < 0.05 significantly different between the immunized group and the DSS group; using one-way ANOVA followed by Dunnett’s *t*-test). **(D,E)** Colon length was measured as an indirect marker of inflammation.

Acute colitis was induced in mice by administering drinking water with 3% (w/v) DSS for 7 days, as previously described (Giner et al., 2016). Animals were randomly assigned to four groups: Control group (mice received only regular drinking water); DSS group (mice received 3% DSS in drinking water); FhEV + DSS group (mice were subcutaneously inoculated three times with *F*. *hepatica* EVs resuspended in PBS, and received 3% DSS in drinking water); and FhEV group (mice subcutaneously inoculated three times with *F*. *hepatica* EVs, and received regular drinking water) (see **Figure 1B**). At day 49 mice were sacrificed, their colons were removed and samples analyzed. Every effort was made to minimize animal suffering and reduce the number of animals used.

### Disease Activity Index

Disease Activity Index was used to assess the severity of colitis as indicated by Giner et al. (2013). Mice were checked daily for development of colitis by monitoring body weight, fecal occult blood (Hemoccult^®^ II Sensa; Beckman Coulter), or gross rectal bleeding, and stool consistency. Overall disease severity was assessed by a clinical scoring system defined as follows: weight loss: 0 (no loss), 1 (1–5%), 2 (5–10%), 3 (10–15%), and 4 (>15%); stool consistency: 0 (normal), 2 (loose stool), and 4 (diarrhea); and bleeding: 0 (no blood), 1 (Hemoccult^®^ positive), 2 (Hemoccult^®^ positive and visual pellet bleeding), and 4 (gross bleeding, blood around anus).

### Histological Analysis

Distal colon parts were cut and fixed as previously reported (Giner et al., 2016). Five-micrometer tissue sections were stained with hematoxylin and eosin and evaluated using an Optiphot Nikkon, microscope by an expert pathologist (CM). A well-accepted histology score in an scale of 0 to 6 was used (Melgar et al., 2008) (0 = no signs of damage; 1 = few inflammatory cells, no signs of epithelial degeneration; 2 = mild inflammation, few signs of epithelial degeneration; 3 = moderate inflammation, few epithelial ulcerations; 4 = moderate to severe inflammation, ulcerations in more than 25% of the tissue section; 5 = moderate to severe inflammation, large ulcerations of more than 50% of the tissue section; and 6 = severe inflammation and ulcerations of more than 75% of the tissue section). In addition, the precise percentage of ulcerated mucosa in every transversal section of the colon was recorded. Median histopathology score and median percentage of ulcerated mucosa were used for comparison between groups.

### Cytokine Production in Tissue

TNF-α, IL-6, IL-17A, and IL-10 concentrations were measured as previously described (Giner et al., 2016), by using specific enzyme immunoassay kits, following manufacturer’s instructions. Reads were done in an iMark^TM^ microplate absorbance reader (Bio-Rad, CA, United States). Values of cytokines were expressed as pg per mg of total protein.

### Determination of Neutrophil Infiltration in Colon Tissue

Neutrophil infiltration was determined by assaying MPO activity, as previously described (Giner et al., 2011, 2013). Absorbance was measured spectrophotometrically at 630 nm, and MPO activity was expressed as the amount of enzyme necessary to produce a change in absorbance of 1.0 Unit g^-1^ of tissue (Recio et al., 2000).

### Cytosolic and Nuclear Protein Extraction

The differential extraction of proteins from intestines and their concentration were determined as previously reported (Giner et al., 2011).

### Western Blot Analysis

Equal amounts of protein (25 μg) were separated by SDS-PAGE in 10% polyacrylamide gels, transferred onto nitrocellulose membranes, blocked, and incubated overnight at 4°C with anti-COX-2 (1:8000), anti-p65 NF-κB (1:500) subunit, or anti-GAPDH, anti-p38 MAPK, anti-Pp38 MAPK (all at 1:1000). Blots were washed with TBS (10 mM Tris-HCl pH 7.4, NaCl 150 mM), incubated with secondary antibodies (1:10000), and immune reactive bands were visualized with the aid of Amersham^TM^ ECL Select western blotting system (GE Healthcare, Madrid, Spain) (Marcilla et al., 1995; Giner et al., 2013).

To unify Western blot densitometry to result in the processed images, data from the DSS group were taken as reference and assigned the value of 100. Relative percentages of the other groups were then calculated.

### Statistical Analysis

The results are expressed as the mean ± SE values. Statistical significance was determined with a one-way analysis of variance (ANOVA), and Dunnett’s *t-*test for multiple comparisons using GraphPad Prism, version 6 (GraphPad Software Inc., La Jolla, CA, United States). Values of *p* < 0.05 were considered to be statistically significant, and the symbols ^∗^, ^#^ and ^+^ were used to indicate the statistical significance.

### Software

Images for all Western blot experiments were acquired with a ChemiDoc MP imager (Bio Rad, CA, United States). Digital images were processed and band density measurements were made with the aid of NIH Image J software (Schneider et al., 2012).

## Results

### Preventive Treatment With *F. hepatica* EVs Ameliorates Clinical Symptoms and Attenuates Histological Alterations in DSS-Induced Acute Colitis

Previous evidence suggests that EVs from parasitic helminths could be potential new therapeutic alternatives against autoimmune diseases (Buck et al., 2014; Montaner et al., 2014). We therefore assessed their putative preventive effect in IBD. EVs were purified from *F. hepatica* parasites cultured as described previously (Marcilla et al., 2012), showing to be a clean and homogeneous preparation, with most vesicles ranging in the size of 50–100 nm (**Figure 1A**).

To test whether FhEVs could prevent colitis, C57BL/6 mice were injected subcutaneously with 10 μg of FhEVs/mouse/injection on days 0, 15, and 30 before colitis induction by DSS at day 42 (**Figure 1B**). We used these amounts of EVs based on previous studies (Trelis et al., 2016). FhEVs-treated mice were considerably less susceptible to DSS-induced colitis and lost around 10% of their initial body weight, whereas non-treated mice lost around 20% after 7 days of DSS treatment (data not shown). Accordingly, DAI of FhEVs-treated colitic mice was lower than in colitic mice on day 5 after the initiation of DSS treatment. Moreover, this difference gradually increased over time (**Figure 1C**). On autopsy, a significant colonic shortening was detected in the DSS-administered groups as compared to the water-administered ones injected or not with FhEVs. However, between DSS-treated mice, FhEVs injected mice had significant larger colons than their non-injected counterparts (**Figures 1D,E**).

### FhEVs Attenuate Histological Alterations and Suppress Neutrophil Infiltration Upon DSS Challenge

**FIGURE 2:**
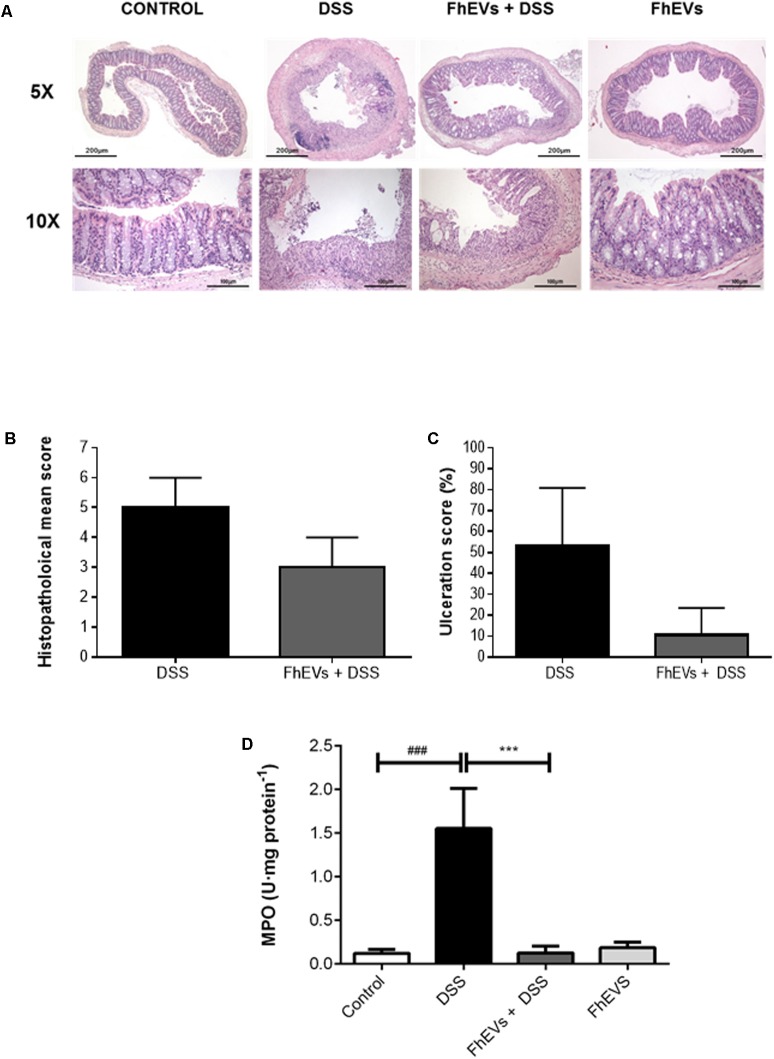
Histopathological changes in the colons of acute colitis mice. **(A)** Distal colon tissue samples were examined using haematoxylin-eosin staining (5× and 10×). **(B)** Histopathological score of colon tissue samples as a mean histopathology score in DSS and FhEVs+DSS mice. **(C)** Percentage of ulcerated colonic mucosa in DSS and FhEVs+DSS mice. **(D)** Myeloperoxidase (MPO) activity was determined by a spectrophotometric method and expressed as the amount of enzyme necessary to produce a change in absorbance of 1.0 unit/mg of tissue. Each bar chart represents the mean ± SEM for at least three independent experiments (*n* = 3 animals) (^###^*p* < 0.001, significantly different from the DSS group vs. control group; ^∗∗∗^*p* < 0.001 significantly different between the DSS group and the group immunized with FhEVs; using one-way ANOVA followed by Dunnett’s *t*-test).

Histologic analyses of colonic sections revealed complete disruption of the colonic architecture in colitic mice, whereas FhEVs-treated colitic mice display a better preservation of tissue architecture and reduced epithelial denudation, crypts distortion, and leukocyte infiltration of the lamina propria (**Figure 2A**), thus resulting in a lower histopathological mean score in this group (**Figure 2B**). Consistent with the histological findings, the ulceration score of FhEVs-treated colitic mice was significantly lower than in DSS-induced colitic mice (**Figure 2C**).

Furthermore, the reduction in neutrophil infiltration observed by histological inspection in FhEVs-treated colitic mice was confirmed by the reduced MPO activity detection in colon tissues from these mice (**Figure 2D**). Altogether, these results strongly support the view that injection of FhEVs could ameliorate DSS-induced murine experimental colitis.

### FhEVs Decrease Pro-inflammatory Cytokines in Colon Tissues

**FIGURE 3:**
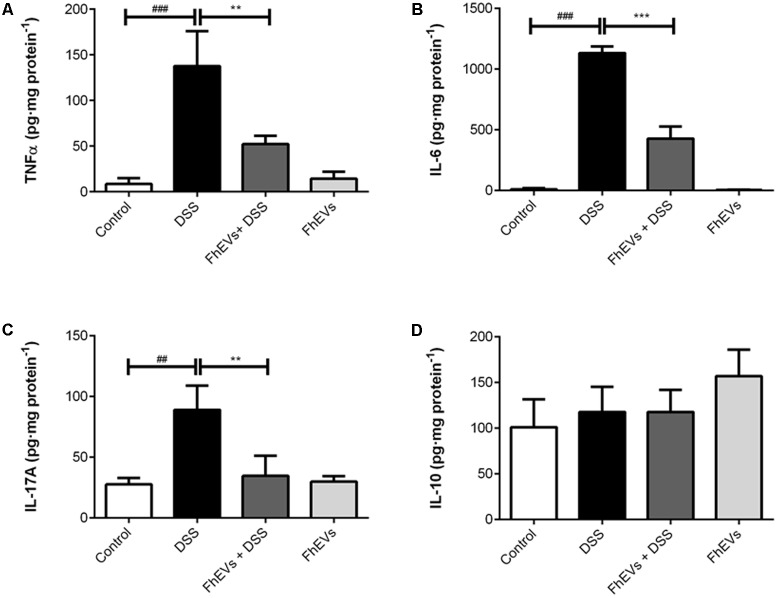
Effect of FhEVs on the profile of cytokine levels in colon tissue samples. At day 49 cytokine levels in colon homogenates were determined by ELISA. The amount of cytokines was expressed as pg per mg of protein. **(A)** TNFα levels, **(B)** IL-6 levels, **(C)** IL-17A levels, **(D)** IL-10 levels. Each bar chart represents the mean ± SEM for at least three independent experiments (*n* = 3 animals). ^###^*p* < 0.001 significantly different from the DSS and control for TNFα and IL6; ^##^*p* < 0.01 significantly different from the DSS and control for IL17A; ^∗∗∗^*p* < 0.001 significantly different from the DSS and immunized group for IL6; ^∗∗^*p* < 0.01 significantly different from the DSS and immunized group for TNFα and IL6, using one-way ANOVA followed by Dunnett’s *t*-test.

To address the effect of FhEVs in colitis we next analyzed the immune response involved by determining the tissue levels of cytokines known to participate in IBD (Giner et al., 2016). Amounts of TNF-α, IL-6, IL-17A, and IL-10 were determined in intestine by ELISA. As shown in **Figures 3A,B**, treatment with FhEVs markedly prevented the increase in the levels of the pro-inflammatory cytokines TNF-α and IL-6 observed in the intestine of DSS-treated mice.

Recent studies have identified that the expression of genes involved in Th17 immune response can distinguish patients with ulcerative colitis from patients with Crohn disease (Rosen et al., 2017). To address whether Th17 response was also modified in the mice model of ulcerative colitis, IL-17A levels in intestine were determined by ELISA. As shown in **Figure 3C**, IL-17A levels were lower in FhEVs-treated colitic mice in comparison to DSS-induced colitic mice, indicating the anti-inflammatory effect of FhEVs. As a parameter of the anti-inflammatory activity of regulatory T lymphocytes (Treg), IL10 was also monitored. Interestingly, the amount of this cytokine did not change significantly in the groups of mice analyzed (**Figure 3D**). Taken together these results indicated that *F. hepatica* EVs exerted their preventive effect by altering the pro-inflammatory effect of DSS, not depending of a role of Treg cells.

### FhEVs Decrease the Activation of the Pro-inflammatory Effector Molecules COX-2, NFkB, and Phosphorylated p38 MAPK in DSS-Induced Acute Colitis

**FIGURE 4:**
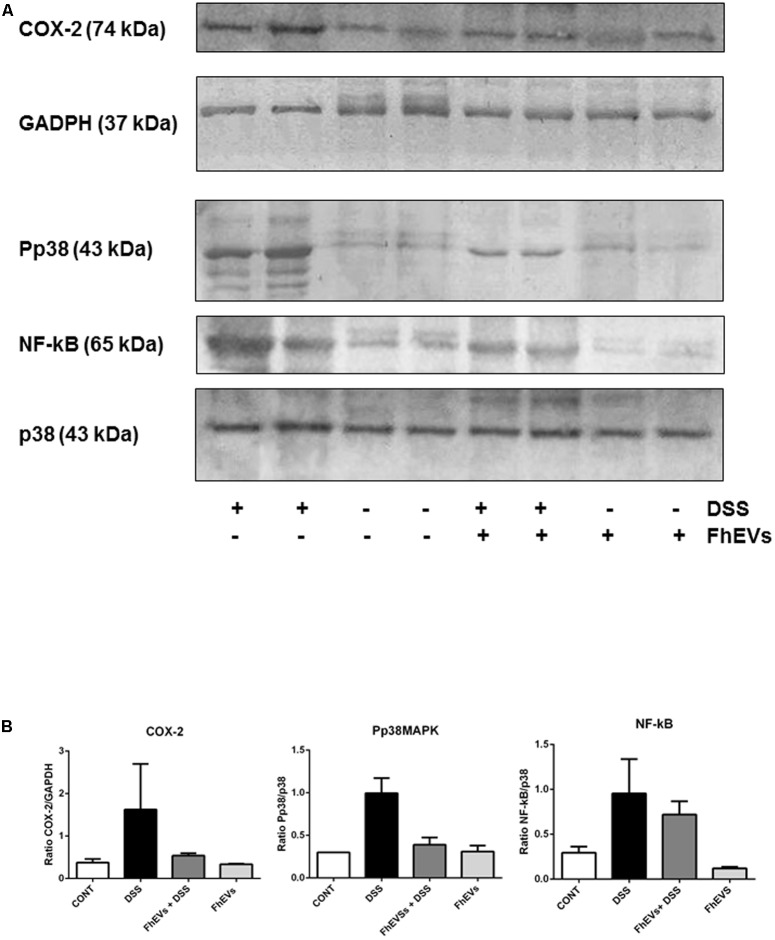
Effects of FhEVs on COX-2 and NF-kB expression, and phosphorylation of p38MAPK (pp38) in DSS-induced acute colitis in mice. At day 49 colon tissues were powdered in a mortar with liquid nitrogen, and tissue proteins were extracted. **(A)** Representative western blot analyses are shown (from three different experiments, *n* = 3). The amount of COX-2 was determined by densitometry analysis, and normalized to GAPDH content for each sample. The amount of phosphorylated p38MAPK (pp38MAPK) and p65 NF-kappaB were determined by densitometry analysis, and normalized to p38MAPK content. Quantification measures were done using NIH ImageJ software (Schneider et al., 2012), considering the amount of protein in DSS group as 100%. **(B)** The histograms representing the data derived from representative western blots following densitometry analysis of each group are also shown.

It is well known that several key factors participate in the inflammatory cascade leading to colitis (Talero et al., 2008; Giner et al., 2011). In this context, different studies have documented the role of COX-2 in mediating the prolonged epithelial secretion, and the barrier dysfunction observed in colonic inflammation in mice (Zamuner et al., 2003; Sanchez-Fidalgo et al., 2013). To address whether *F. hepatica* EVs administration could also affect the expression of COX-2, as pro-inflammatory mediator in DSS-induced colitis, we next analyzed its levels in colon tissue by western blot. As shown in **Figure 4**, the DSS-induced colitic mice showed higher levels of COX-2 in colon tissues than their FhEVs-pretreated counterparts (only 33% of COX-2 was detected as compared to the levels observed in DSS-induced colitic mice, **Figure 4B**).

Furthermore, colonic cells showed variations in different signaling pathways as in the mitogen-activated protein kinase (p38 MAPK), and NF-kB pathways. As shown in **Figure 4**, DSS increased the phosphorylation of p38 MAPK, but pre-treatment of colitic mice with FhEVs produced lower levels of phosphorylation of p38 MAPK (around 39%), although still higher than in colon from control and FhEVs-injected mice groups (both without DSS administration) (**Figure 4B**).

The levels of NF-kB in the nuclear fraction of colon cells were also quantified, and colon tissues from both DSS-induced colitis mice contained higher levels of this nuclear factor than controls. Nevertheless, FhEVs treated colitic mice contained around 25% less amount of NF-kB when comparing to DSS-induced colitic mice (**Figures 4A,B**).

Altogether, our results indicate that *F. hepatica* EVs interfere with signaling pathways involved in acute ulcerative colitis promoted by DSS, and suggest their role in preventing pro-inflammatory cascades in which these key molecules participate.

### The Preventive Effect of FhEVs in Colitis Is Not Mediated by Lymphocytes

**FIGURE 5:**
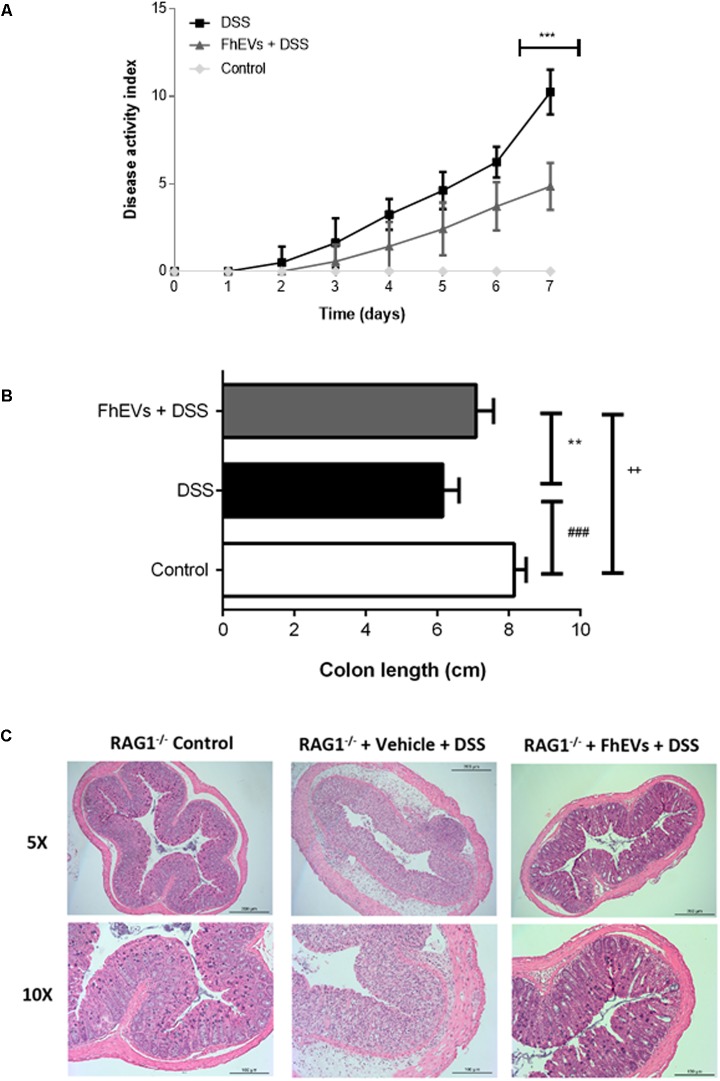
Lymphocytes do not mediate the preventive effect of FhEVs in DSS-induced colitis. **(A)** Disease activity index (DAI) evaluated daily combining the parameters of weight loss, diarrhea, and bleeding in Rag1^-/-^ mice (*n* = 7 animals) as described in methods. Statistical analyses were performed using a one-way ANOVA followed by Dunnett’s multiple comparison *post hoc* test (^∗∗∗^*p* < 0.001, FhEVs+DSS versus DSS). **(B)** Colon length was measured as an indirect marker of inflammation. Statistical significance between two experimental groups was assessed using the Independent-Sample *t*-test (^###^*p* < 0.001, significantly different between the control and DSS group; ^∗∗^*p* < 0.01 significantly different between the immunized group and the control; ^++^*p* < 0.01 significantly different between the immunized group and the DSS group; using one-way ANOVA followed by Dunnett’s *t*-test). **(C)** The histopathological changes of colons in mice were examined using H&E staining (5× and 10×).

To assess whether lymphocytes were the cells accounting for the protection, similar *in vivo* experiments of immunization with FhEVs before DSS administration were carried out in Rag1^-/-^ mice. These mice lack lymphoid B and T cells (Mombaerts et al., 1992), and are susceptible to DSS-induced colitis (Kim et al., 2006; Kiesler et al., 2015). Rag1^-/-^ mice were injected with FhEVs subcutaneously. Interestingly, FhEVs exhibited protection against the chemical treatment (**Figure 5**), similarly to what happened in C57BL/6 mice (see **Figures 1, 2**).

*Fasciola hepatica* EVs-treated mice lost around 9% of their initial body weight, whereas non-treated mice lost around 18%, after 7 days of DSS treatment (data not shown). Accordingly, the DAI of FhEVs-treated colitic mice was lower than in colitic mice on day 4 after the initiation of DSS treatment, increasing this difference over time (**Figure 5A**). DSS-administered mice treated with FhEVs had significant larger colons than their non-injected counterparts, although not reaching the length observed in control animals (**Figure 5B**).

When histological analyses of colonic sections were performed, they revealed large disruption areas of the colonic architecture in colitic mice, whereas FhEVs-treated colitic mice displayed preservation of tissue architecture and reduced epithelial denudation and crypts distortion (**Figure 5C**). Altogether, these results supported the notion that neither T nor B cells are involved in the preventive effect observed with the parasite EVs.

## Discussion

Intestinal bowel disease is one of the most important diseases affecting developing countries, and it is currently spreading in undeveloped ones (Molodecky et al., 2012; Ng et al., 2013). The etiology of the disease is not well understood, with a complex pathology which has no effective treatment available. In fact, the development of effective treatments require studies using multidisciplinary approaches, where animal models have proven highly useful (Lin and Hackam, 2011). DSS-induced colitis model has been widely used to explore new therapeutic options (Wirtz and Neurath, 2007; Kiesler et al., 2015).

Among the new treatments, the use of biologicals is gaining attention (Rogler, 2015; Maizels, 2016). In this context, and following the Hygiene hypothesis (Strachan, 1989), clinical trials with parasitic helminths have been initiated, and some are underway (Smallwood et al., 2017). Some concerns about using helminths to treat IBD have arisen, with the possibility of producing malignancy (Bonovas et al., 2016). In fact, a recent report has shown that the treatment with the parasitic nematode *Heligmosomoides polygyrus* can induce tumor progression in a DSS-induced colitis in Balb/c mice (Pastille et al., 2017).

Along with those potential clinical problems, the use of parasites to treat IBD also face ethical problems (McSorley and Maizels, 2012), so the alternative of using parasite defined products, like excretory/secretory products (ESP), or isolated molecules seem to be a good option. In this context, studies with a crude extract from the laminated layer of *Echinococcus granulosus* (a tapeworm parasite) showed not only its preventive effect but also played a beneficial role in maintaining the integrity of the intestinal mucosal barrier against DSS-induced injury (Soufli et al., 2015). Those results supported the role of EVs as promoters of the epithelial barrier function (Xu et al., 2016).

Previous studies had shown that EVs administered either orally or injected could reach the colon of mice and exert their therapeutic effect on induced colitis (Ju et al., 2013; Yang et al., 2015). Very recently, Wang et al. (2017) have reported a protective effect in a similar mice model when using EVs produced by dendritic cells previously exposed to eggs from the parasitic trematode *Schistosoma japonicum* (Wang et al., 2017). In this regard, our data highlight that the parasite *F. hepatica* EVs have a potent modulatory effect on the immune response in DSS-induced colitis in mice. We provide evidence that *F. hepatica* EVs can prevent DSS-induced colitis by down regulating pro-inflammatory cytokines like TNFα, IL-6, and IL17A, as well as suppressing MAPK/NF-κB signaling pathways. Accordingly, FhEVs treatment decreased MPO activity, indicating a reduction in neutrophil infiltration in damaged colon and which has also been corroborated in the histological sections. In accordance, reduce levels of cytokines TNF-α and IL-17A have been detected in the colon of FhEVs-treated mice, which are inducers of neutrophil transmigration (Kolls and Lindén, 2004; Griffin et al., 2012). DSS colitis can be exacerbated by granulocyte recruitment (Natsui et al., 1997; Williams and Parkos, 2007; Sander et al., 2008; Chami et al., 2014; Kishida et al., 2015). The activity of many enzymes and chemicals produced by neutrophils is not specific to pathogens, so they can damage host tissues when released extracellularly, contributing to the aggravation of mucosal inflammation. Likewise, in UC, unrestricted neutrophil activation may cause significant tissue damage that further leads to chronic pathology and the extent of neutrophil infiltration correlates with the severity of the disease (Bressenot et al., 2015). Therefore, the reduced neutrophil infiltration in FhEVs-treated mice, would provide for the protection effect detected in this model of acute colitis induced by DSS, and may be crucial for the treatment of UC patients. In Rag1^-/-^ mice, a decreased neutrophil infiltration is also observed in the histology sections after FhEVs treatment, in accordance with the results obtained in C57B/L6 mice. It is important to remark that though Rag1^-/-^ mice have no mature T and B lymphocytes (Mombaerts et al., 1992), develop totally normal granulocytes, like WT mice, as has been demonstrated in reports which use these mice in infection models (Smith et al., 2009; Seymour et al., 2015; Carey et al., 2016; Papp et al., 2016).

Furthermore, our results suggest that neither lymphocytes nor IL-10 are involved in the protective effect by FhEVs in DSS-induced colitis mice model. This seems to be at odds with previous reports showing that either splenic B cells or EVs from granulocytic myeloid-derived suppressor cells, attenuate DSS-induced colitis by promoting Tregs expansion, and inhibit Th1 cells proliferation (Reyes et al., 2015; Wang et al., 2016).

The accurate identification of the molecules responsible for the anti-inflammatory effect is underway, but suitable candidates are molecules previously identified in FhEVs, like FABPs (Cwiklinski et al., 2015), which have been shown to induce anti-inflammatory response in animal models by diminishing the levels of TNFα (Ramos-Benitez et al., 2017). Other exosome-contained molecules like miRNAs represent potential candidates as they may act as immunomodulators of the intestinal innate immune response (Buck et al., 2014). Interestingly, a repertoire of miRNAs with immune-regulatory function have been found in *F. hepatica* EVs previously by our group (Fromm et al., 2015).

Resident intestinal macrophages and colonic dendritic cells have been reported to have a high anti-inflammatory phenotype and are hypo-responsive to microbial stimuli in rodents and humans (Bilsborough and Viney, 2004; Kelsall and Leon, 2005; Smith et al., 2005). In line with this, it has been described that depletion of both dendritic cells and macrophages from the intestinal lamina propria in C57BL/6, BALB/c, and SCID mice (without T and B cells) increased DSS colitis severity. These mice had increased neutrophilic inflammation, epithelial injury, and enhanced mucin depletion from globet cells (Qualls et al., 2006). On the other hand, helminth infections are associated with the induction of immunosuppressive M2/Alternatively Activated Macrophages (AAMs) (Loke et al., 2000; Herbert et al., 2004). It is therefore conceivable that FhEVs are enhancing the anti-inflammatory functions of these innate cell populations as they are protective in Rag1^-/-^ mice that lack T and B cells populations. Other helminth infections have been shown to prevent colitis development independently of Tregs stimulation, through the inhibition of prostaglandins by AAMs (Ledesma-Soto et al., 2015) or recruitment of a novel macrophage population distinct to AAMs or Gr1^+^ macrophages (Smith et al., 2007). Moreover, in addition to their immunosuppressive role, lamina propria macrophages play an important role in the regeneration of damaged epithelium by controlling the epithelial progenitor niche after DSS-induced colitis (Pull et al., 2005). On the other hand, the reduced neutrophil infiltration detected in FhEVs-treated mice might be a consequence of the decreased epithelial barrier damage and diminished production of pro-inflammatory cytokines from the resident myeloid cell populations in the gut as observed in the foregoing reports, because of the preventive role of these exosomes. Moreover, some reports described that subcutaneously injected exosomes can be detected in several organs including the gastrointestinal tract even after 24 h of administration, and cleared from the bloodstream in a few minutes (Wiklander et al., 2015). However, in our experimental settings the exosomes are administered 12 days before DSS administration, which makes us to think that they are exerting their role in the gut resident populations. A plausible mechanism may be epigenetic reprogramming of these innate cell populations, a process that has been recently termed as trained immunity (Netea et al., 2016), or even of the intestinal epithelial cells. Whether, in addition, FhEVs could be exerting their function by altering granulopoiesis at the bone marrow level, or influencing in endothelial cells the level of expression of adhesion molecules, requires further experimentation and biodistribution analyses.

Apart from the induction of strong Th2 responses and IgE production, which can be discarded in our experimental setting due to the maintenance of the protective effect in Rag1^-/-^ mice that lack mature T and B lymphocytes, helminths also promote the expansion of eosinophils, mast cells and basophils which could not be discarded. Levels of these cell populations can be measured by flow cytometry to evaluate if there were differences among FhEVs-treated versus non-treated mice. Interestingly, among these cell populations, the only one which have been described to promote a protective role in acute DSS colitis are the eosinophils, due to the production of anti-inflammatory lipid mediators (Masterson et al., 2015). To further elucidate which innate cell population is mediating the protection, adoptive transfer experiments could be performed by injecting specific sorted cell populations derived from FhEVs-immunized Rag1^-/-^ mice into non-immunize ones. Our most likely candidates are macrophages, as stated in the foregoing works. Besides, macrophages are highly phagocytic cells that could be acquiring a highest number of exosomes. Moreover, the role of dendritic cells is typically focused on T cell polarization, which are not involved. Specific deletion of macrophages can be performed by the use of liposomal clodronate agent. If they turn to be involved in the conferred protection to DSS-colitis, it would be very informative to evaluate the role of FhEVs in M1 and M2 polarization, which could be performed by flow cytometry or RNA-seq.

Our findings suggest that other immune cells aside lymphocytes are involved in the protective response (Masterson et al., 2015; Coakley et al., 2017; Wang et al., 2017; Campbell et al., 2018). Future studies will address the identification of the immune cells involved in the FhEVs protective effect and the mechanisms behind it. The fact that the conferred protection against intestinal inflammation is mediated by EVs is of great importance to move this research forward into translational applications.

## Author Contributions

AM, MT, DB, MR, RG, and FS-M: participated in the study concept and design. JR, AG, MS, FC, CM, DB, and MT: acquisition of data. JR, AG, MS, EG, CM, MR, RG, DB, FS-M, and AM analyzed and interpreted data. MR, RG, DB, FS-M, and AM drafted and critically revised the manuscript.

## Conflict of Interest Statement

The authors declare that the research was conducted in the absence of any commercial or financial relationships that could be construed as a potential conflict of interest. The reviewer PX and handling Editor declared their shared affiliation.

## Abbreviations

COX-2: cyclooxygenase 2
DAI: disease activity index
DSS: dextran sulfate sodium
EVs: extracellular vesicles
FABPs: fatty acid-binding proteins
Fh: *Fasciola hepatica*
IBD: inflammatory bowel disease
IL: interleukin
MAPK: mitogen activated protein kinase
MPO: myeloperoxidase
NF-kB: nuclear factor kappa B
TEM: transmission electron microscopy
TNF-α: tumor necrosis factor alpha
